# Optimal application of soft-palate webbing flap pharyngoplasty combined with nasal surgery for surgical treatment of primary snoring and obstructive sleep apnea

**DOI:** 10.1007/s11325-022-02563-9

**Published:** 2022-02-05

**Authors:** Jin-A Park, Hyunkyung Cha, Su Keun Kim, Hyunjun Woo, Seung Cheol Han, Do Won Kim, Doo Hee Han, Dong-Young Kim, Chae-Seo Rhee, Hyun Jik Kim

**Affiliations:** Department of Otorhinolaryngology, Seoul National University College of Medicine, Seoul National University Hospital, Seoul, Korea

**Keywords:** Obstructive sleep apnea, Soft-palate webbing flap pharyngoplasty, Primary snoring, Surgical indications

## Abstract

**Background:**

Excessive collapse of the soft palate and lateral pharyngeal wall narrowing are established causes of loud snoring and sleep apnea in subjects with obstructive sleep apnea (OSA). Therefore, delicate surgical techniques are needed to reshape the soft palate and create sufficient tension in the lateral pharyngeal wall. This study aimed to determine the therapeutic outcome and favorable indications of soft-palate webbing flap pharyngoplasty in subjects with OSA and primary snoring.

**Methods:**

A total of 174 subjects who underwent soft-palate webbing flap pharyngoplasty combined with uvulopalatal flap and septoturbinoplasty from August 2015 to February 2020 were included in this study. Medical records, including pre- and postoperative sleep parameters, were retrospectively reviewed. The primary outcome measure was the degree of improvement in AHI after surgery. Other outcomes were differences in surgical response rates, subjective visual analog score (VAS) for snoring, sleep quality, and complications.

**Results:**

Polysomnographic results showed that apnea–hypopnea index (AHI) scores were significantly reduced from 39.6 ± 6.1 to 22.9 ± 3.6 following soft-palate webbing flap pharyngoplasty in 59 subjects, and overall success and response rates of this technique were analyzed with 71%. We found that the successful outcomes were observed in 50% of mild (n = 12) and 56% of moderate (n = 16) subjects with OSA subjects due to lateral pharyngeal wall collapse. The success rate of soft-palate webbing flap pharyngoplasty was relatively higher in subjects with mild and moderate OSA than those with severe OSA. Additionally, the mean VAS snoring scale was 4.7 and subjects’ primary snoring intensity significantly improved to 2.9 after soft-palate webbing flap pharyngoplasty. Subjective symptoms such as daytime sleepiness and sleep quality also showed improvement. Most complications were found to be minimal and improved by 1 month after the operation.

**Conclusion:**

Our data demonstrate that soft-palate webbing flap pharyngoplasty is an effective treatment for OSA and primary snoring and may be a promising technique to reduce lateral pharyngeal wall collapse.

## Introduction

Based on previous studies, repeated narrowing of the upper airway is pathogenetic mechanism in obstructive sleep apnea (OSA) and subjects with OSA suffer from limited airflow from the nasal cavity. Upper airway narrowing predisposes the pharyngeal wall to collapse due to increased negative pressure, faster airflow, and higher airway resistance [1, 2]. More collapsible airway causes sleep-related symptoms, such as loud snoring and apneic events, and leads to fatigue, daytime sleepiness, and systemic complications if those symptoms are not properly managed in subjects with OSA [3–8]. The upper airway narrowing occurs at multiple structures, including the soft palate, uvula, palatine tonsils, lateral pharyngeal walls, and the tongue base [6, 7]. In particular, the lateral wall of oropharynx is composed of several muscular structures, such as the palatopharyngeus, superior pharyngeal constrictor, and palatoglossus muscles, in addition to the palatine tonsils and lymphoid tissues around soft palate. Retropalatal circumferential narrowing due to lateral pharyngeal wall collapse has been documented to be a critical structural cause of OSA but so far, its clinical significance has been underestimated in subjects undergoing sleep surgery [11]. Combined oropharynx, lateral pharyngeal wall, and velum obstruction are considered the most dominant anatomic characteristics of OSA, and the lateral pharyngeal wall is more collapsible or thicker in subjects with severe OSA than in normals or those with mild OSA [12]. Actually, complete lateral pharyngeal wall narrowing may be closely related to higher apnea–hypopnea index (AHI) scores, and excessive lateral pharyngeal collapsibility is seen in subjects with OSA who show a relapse of snoring or apneic events after surgery [13, 14]. Previous clinical research has demonstrated the clinical benefits of palatal surgeries for OSA with lateral pharyngeal wall collapse, including relief of subjective symptoms and improvement of sleep parameters [8–10], and diverse surgical techniques such as lateral pharyngoplasty, relocation pharyngoplasty, and expansion sphincter pharyngoplasty (ESP) to improve lateral pharyngeal wall narrowing and intensify the stability of the lateral pharyngeal wall have been introduced [13–18].

The posterior palatal pillars have excessive mucosa tissue (webbing) with significant redundancy, which is an important anatomical structure that contributes to increase of lateral pharyngeal wall collapse. Soft-palate webbing flap pharyngoplasty has recently been introduced to reshape the soft-palate webbing without tonsillectomy [19, 20]. Soft-palate webbing flap pharyngoplasty may offer good benefits for creating stability in the lateral pharyngeal walls resulting in the reduction of the number of apneic events or snoring intensity without considerable postoperative pain.

In this study, we aimed to evaluate the therapeutic outcomes of soft-palate webbing flap pharyngoplasty in subjects with OSA or primary snoring and to determine the favorable indications of this procedure in the subjects with lateral pharyngeal wall collapse.

## Material and methods

### Study design

A total of 174 subjects who had been diagnosed with OSA at the Department of Otorhinolaryngology of Seoul National University Hospital from August 2015 to February 2020 were included in this study. OSA was diagnosed using overnight polysomnography (PSG, Glael 4 K, Compumedics, Victoria, Australia), and respiratory events such as apnea and hypopnea were scored as defined in the AASM scoring manual version 2.6. All subjects underwent soft-palate webbing flap pharyngoplasty, uvulopalatal flap procedure, and septoturbinoplasty to relieve their sleep-related symptoms and to improve abnormal sleep parameters [21]. Our indications for soft-palate webbing flap pharyngoplasty were: (1) AHI (events/hr) > 5 events/hr on PSG; the severity of OSA was defined as mild for an AHI ≥ 5/h and < 15/h, moderate for an AHI ≥ 15/h and ≤ 30/h, and severe for an AHI ≥ 30/h, (2) retropalatal circumferential narrowing above the drug-induced sleep endoscopy (DISE) grade I (> 50% narrowing) with reference to the VOTE classification [22]; (3) lateral pharyngeal wall narrowing with the bulky redundant soft tissue around the posterior pillar; and (4) bilateral tonsil hypertrophy < grade II. We recommended this pharyngoplasty to patients with primary snoring (AHI < 5). DISE was performed in 174 subjects prior to soft-palate webbing flap pharyngoplasty, and subjects who fit the indication of soft-palate webbing flap pharyngoplasty were recruited. All subjects also complained of nasal obstruction due to septal deviation with bilateral inferior turbinate hypertrophy and septoturbinoplasty were performed together with soft-palate webbing flap pharyngoplasty.

**Fig. 1:**
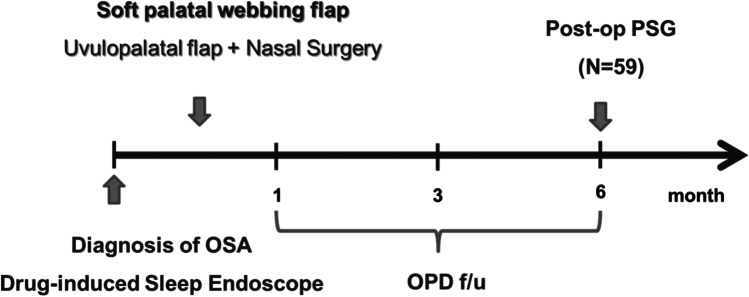
Schematic figure of the study design and clinical evaluation

The 59 subjects who underwent PSG prior to and after soft-palate webbing flap pharyngoplasty and medical records, including results from pre- or postoperative PSG, were retrospectively reviewed. Total sleep time, AHI, and lowest O_2_ saturation were observed preoperatively and six months postoperative (Fig. 1). A successful outcome was defined as at least 50% reduction in AHI from baseline and AHI < 20, as described by Sher [23]. A successful outcome was also analyzed with “success” being defined as > 50% reduction in AHI and postoperative AHI < 20, and “response” being defined as > 50% reduction in AHI and response rate was defined by adding the ratio of success and response group [24]. Hospital stay duration, early complications, the Epworth Sleeping Scale (ESS), and the visual analogue score (VAS) for snoring (observed by subject’s sleep partner), breathing while awake, and sleep quality were evaluated recorded on hospital discharge and during follow-up visits.

### Surgical technique

OSA subjects were selected for soft-palate webbing pharyngoplasty if they had retropalatal circumferential narrowing according to DISE findings and if they did not present with obstruction at the tongue base or epiglottis. All subjects complained of nasal obstruction, and they were scheduled to receive septoturbinoplasty to improve nasal breathing. The indicated operation was performed under general anesthesia with the subject on the supine position and orotracheal intubation.

**Fig. 2:**
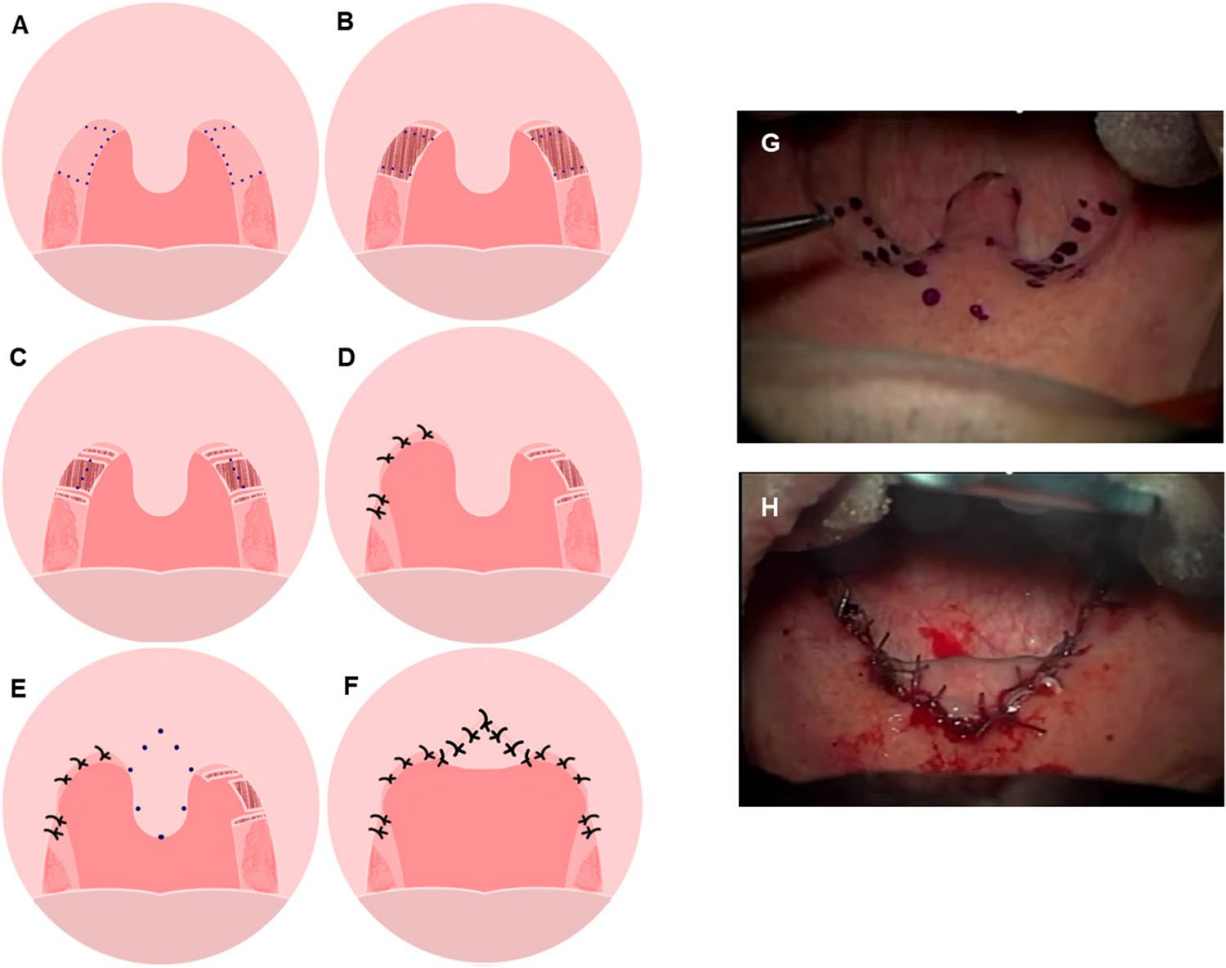
Main surgical steps in soft-palate webbing flap pharyngoplasty. (A) Incision line for dissection of soft-palate webbing layers was determined. (B) Dissection of soft-palate webbing layers with removal of the oropharyngeal layer and creation of two flaps; the posterior pillar flap and the soft-palate webbing flap were placed laterally. (C, D) Dissected nasopharyngeal layer of palate webbing, turned up to cover the free edge of the newly formed soft palate and the upper part of the laterally placed posterior pillar flap fixed in place with Vicryl #3.0. (E) The same procedure is performed on the other side. (F) Uvulopalatal flap was carried out as the last step of the soft-palate webbing flap pharyngoplasty procedure and mucosal closure was performed. Pre- (G) and postoperative (H) findings of soft-palate webbing flap pharyngoplasty

The surgical technique used for soft-palate webbing flap pharyngoplasty in this study was based on a method originally developed by Elbassiouny et al. [20]. The ventral (oropharyngeal) layer of the palate webbing is dissected from the dorsal (nasopharyngeal) layer starting at the superior margin of the palate webbing moving down and extending the dissection to the mucosa of the posterior pillar to expose the supra-tonsillar area (Fig. 2A). Using blunt dissection in the supra-tonsillar area following retraction of the anterior pillar, the fibers of the palatopharyngeus muscle are exposed up to the lateral boundary. Using a Bovie cautery, the layer of nasopharyngeal mucosa is released on both sides and then, separate it from the uvula, medially, and the posterior pillar, laterally (Fig. 2B). Two flaps are now available: the lateral flap, which represents the posterior pillar down to the lateral release incision, and the free webbing flap created by release incisions. The dorsal mucosal layer of the palate webbing is shortened to 4–5 mm in length. The upper part of the laterally placed posterior pillar flap is sutured with a Vicryl 3.0, making appropriate tension in the lateral pharyngeal wall and maximally lateralizing the tonsil, which consequently increases the oropharyngeal transverse diameter (Fig. 2C). The shortened dorsal mucosal layer is turned up to cover the free edge of the soft palate and then fixed with same suture material, so that the knob is facing the nasopharynx (Fig. 2D). Now, the free edge of the newly formed soft palate is covered by normal palatal mucosa (Fig. 2E). The same procedure was repeated on the other side.

Next, the uvulopalatal flap was performed indicating the design. Only the uvular mucosa was carefully dissected with a Bovie cautery, and the uvula was sutured using Vicryl 3.0 and fixed onto the soft palate (Fig. 2F). The tension of the suture on the flap was adjusted to prevent postoperative wound dehiscence compared to preoperative findings (Fig. 2G, H). All subjects were discharged one day after undergoing soft-palate webbing flap pharyngoplasty, and the last follow-up visit was performed six months after surgery.

### Statistical analyses

Statistical calculations were performed using SPSS 19.0 (SPSS, IBM, Armonk, NY, USA). Postsurgical changes in AHI, lowest oxygen saturation, ESS value, and VAS for snoring were analyzed using a paired t-test and the Wilcoxon signed-rank test. Descriptive data are presented as means ± standard deviations. Differences are considered significant if *p* < 0.05.

## Results

### Subject demographic data and sleep parameters

We recruited 174 subjects who were diagnosed with OSA and underwent soft-palate webbing flap pharyngoplasty to resolve airway narrowing and reduce lateral pharyngeal collapse without tonsillectomy. The lateral pharyngeal wall narrowing of subjects was confirmed using DISE, and all subjects showed over grade I retropalatal circumferential narrowing. Among them, 149 subjects were men, and 25 were women. Mean subject age was 45.1 years (range 15–73 years), and mean BMI was 25.43 kg/m^2^ (range 19.1–35.57 kg/m^2^). OSA severity was based on AHI: 14 patients had primary snoring, 55 patients had mild OSA, 47 had moderate OSA, and 58 had severe OSA (Table 1). Fourteen patients who underwent soft-palate webbing flap pharyngoplasty were confirmed to have primary snoring. We performed soft-palate webbing flap pharyngoplasty in subjects with OSA who had lower than grade-II tonsils and greater than palatal grade I. Based on preoperative DISE findings, all subjects had circumferential narrowing at the retropalatal level and the mean grade of retropalatal narrowing was 1.6 ± 0.3. Fifty-nine subjects underwent PSG prior to and after soft-palate webbing flap pharyngoplasty, and preoperative PSG findings revealed that the mean AHI was 35.6 ± 22.6 events/hr, mean lowest O_2_ saturation was 80.8 ± 8.0%, mean total sleep time was 368.7 min, and mean REM sleep percentage was 20.1%. Mean operation time was 28.7 ± 5.4 min, and mean admission period was 3.4 days.

**Table 1:** Subject demographic information (N = 174)

Characteristic	Values, No. (%)
Demographics	
Age (years), mean (range)	45.1 (15.0–73.0)
BMI, mean (range)	25.4 (19.1–35.6)
Sex	
Male	149 (86)
Female	25 (14)
Clinical	
AHI, mean (SD), events/h	27.2 (22.4)
OSA severity	
Primary snoring	14 (8)
Mild	55 (32)
Moderate	47 (27)
Severe	58 (33)

BMI: body-mass index, AHI: apnea–hypopnea index, OSA: obstructive sleep apnea

### Therapeutic outcomes from soft-palate webbing flap pharyngoplasty

**Fig. 3:**
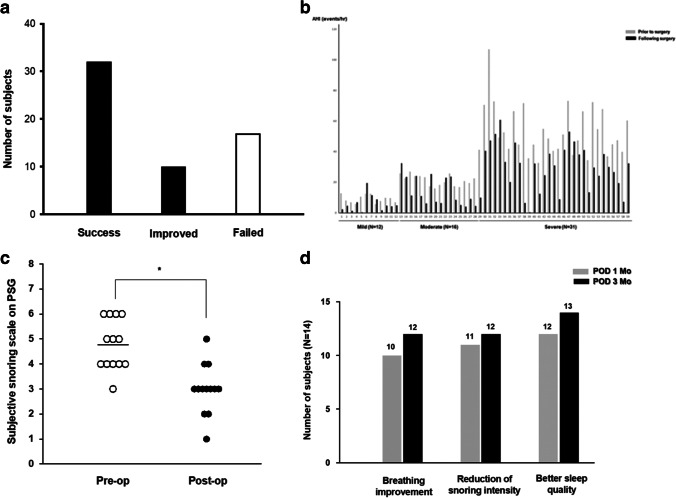
Changes in the sleep parameters of OSA subjects following soft-palate webbing flap pharyngoplasty. (A) The overall success and response rates, with “success” defined as ≥ 50% reduction in AHI and “improved” defined as postoperative AHI reduction ≥ 20, and “failed” defined as < 50% or < 20% AHI reduction. (B) Apnea and hypopnea index of individual OSA subjects who underwent PSG prior to and following soft pharyngeal webbing flap pharyngoplasty. (C) Subjective snoring scale (Scale 1: mild, 2: mild to moderate, 3: moderate, 4: moderate to severe, 5: severe, 6: very severe) and (D) change in subjective symptoms of subjects with primary snoring pre- and postoperatively (N = 14). (*p < 0.05 when comparing grades between primary snoring subjects before and after soft-palate webbing flap pharyngoplasty)

Therapeutic outcomes were evaluated via AHI after soft-palate webbing flap pharyngoplasty, and 59 subjects underwent PSG six months after their operation. The mean AHI for all subjects was 35.6 ± 22.6 prior to soft-palate webbing flap pharyngoplasty. Mean postoperative AHI decreased significantly to 22.9 ± 16.0 among the 59 subjects who underwent PSG six months later. Given an arbitrary selection of 50% reduction in AHI and AHI < 20 as a threshold, 25 subjects responded to their surgery, yielding a sleep-surgery success rate (including soft-palate webbing flap pharyngoplasty) of 42% in patients with OSA and lateral pharyngeal wall narrowing (Table 2). The response rate of soft-palatal webbing flap pharyngoplasty was resulted in 71% and these subjects were assigned to the success and response group (Fig. 3A).

**Table 2:** Comparison of subjective symptoms and polysomnographic parameters before and after soft-palate webbing flap pharyngoplasty (n = 59)

	Preop	Postop	p-value
AHI (h^−1^)	35.6 ± 22.6	22.9 ± 16.0	0.00004
Apnea index (h^−1^)	18.9 ± 18.3	8.4 ± 10.5	0.00006
Supine AHI (h^−1^)	42.8 ± 19.5	18.7 ± 10.2	0.00004
Min SpO_2_ (%)	80.8 ± 8.0	83.3 ± 7.3	0.0044
Time below 90% SpO_2_ (min)	32.3 + 25.5	21.6 + 16.8	0.0032
ODI (h^−1^)	36.4 ± 22.5	20.4 ± 11.4	0.00053
Mean total sleep time (min)	368.7 ± 86.6	403.6 ± 50.9	0.0004
Mean scale of snoring intensity	moderate to severe	mild to moderate	< 0.00008
Mean ESS value	11.3	7.2	0.041
Sleep efficiency (%)	81.0 ± 12.2	85.4 ± 10.0	0.0036
REM percentage (%)	20.1 ± 7.6	21.6 ± 6.3	0.0684

Op: operation, SD: standard deviation, AHI: apnea–hypopnea index, Min SpO_2_: minimal oxygen saturation, ODI: oxygen desaturation index, ESS: Epworth sleepiness scale, REM: rapid eye movement

We classified these 59 subjects according to OSA severity and compared their therapeutic outcomes from soft-palate webbing flap pharyngoplasty according to apneic events. The results showed that the success rate of soft-palate webbing flap pharyngoplasty was 50% in mild OSA (n = 12), 56% in moderate OSA (n = 16), and 32% in severe OSA (n = 31; Fig. 3B). We found that the success rate of sleep surgeries including soft-palate webbing flap pharyngoplasty was relatively higher in the patients with mild and moderate OSA compared with severe OSA.

Postoperative PSG results also showed that the lowest O_2_ saturation level improved significantly from 80.8 ± 8.0% to 83.3 ± 7.3% (*p*-value 0.0044). Additionally, the postoperative mean value of total sleep time was prolonged from 368.7 to 403.6 min (*p*-value 0.0004). For subjective symptoms, mean ESS value reduced from 11.3 to 7.2 (*p*-value 0.041), and subjective sleep quality improved in all 174 subjects (Table 2). Based on PSG findings about snoring intensity, the subjective snoring scale for subjects with primary snoring (n = 14) was also significantly reduced after surgery (Fig. 3C), and subjective symptoms of subjects with primary snoring including breathing, snoring intensity, and sleep quality improved in the six months of postoperative follow-up (Fig. 3D).

**Fig. 4:**
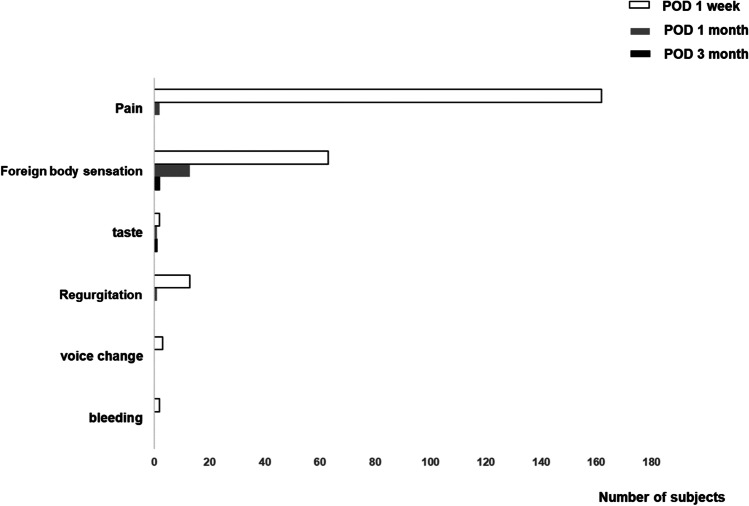
Subjective complaints or complications following soft-palate webbing flap pharyngoplasty. Subjective symptoms: postoperative pain, uncomfortable sensation, taste, VPI, voice change, and bleeding were investigated one week, one month, and six months after soft-palate webbing flap pharyngoplasty (VPI: velopharyngeal insufficiency)

Mucosal wound dehiscence was observed in 2% (n = 4) of subjects after soft-palate webbing flap pharyngoplasty combined with the uvulopalatal flap. We investigated the following postoperative complications: postoperative pain, uncomfortable sensation, taste loss, velopharyngeal insufficiency (VPI), voice change, and bleeding (Fig. 4). We did not observe serious complications related to soft-palate webbing flap pharyngoplasty after one week, one month, or six months. Of 174 subjects, 164 complained of pain one week after soft-palate webbing flap pharyngoplasty, but only two subjects were still experiencing oropharyngeal pain one month after surgery. Just one subject was still experiencing postoperative pain one month later. Additionally, 63 subjects felt an uncomfortable sensation around the soft palate area one week after surgery, but only two subjects complained of it six months postoperatively. A few subjects initially complained of taste loss, VPI, and voice change, but those complaints subsided by the six-month follow-up point. These postoperative complaints were well managed in all patients using a conservative approach. We did not observe postoperative bleeding in any patients at the oropharynx after soft-palate webbing flap pharyngoplasty, and tracheostomy was not performed in any subjects.

## Discussion

The results of this study suggest that soft-palate webbing flap pharyngoplasty may be an effective surgical option for improving lateral pharyngeal wall tension in  subjects OSA, and it provides positive therapeutic outcomes for OSA with retropalatal circumferential narrowing in their upper airway. This surgical technique yielded the best success rates for subjects with mild and moderate OSA or primary snoring if performed together with nasal surgery and our clinical results also suggest favorable surgical indications for subjects who had a narrowed oropharynx due to lateral bulk around the posterior pillar and circumferential narrowing (DISE grade > I) at the retropalatal level.

Subjects with OSA and lateral pharyngeal wall collapse exhibited higher AHI and respiratory disturbance index scores than subjects with anteroposterior narrowing in their upper airway [25–29]. Excessive mucosal thickening around the posterior pillar with significant redundancy could be involved in lateral pharyngeal wall narrowing in subjects with primary snoring and those with OSA. This suggests a need for a surgical option to reduce lateral pharyngeal wall narrowing and adequate reduction of redundant tissue around the posterior pillar to provide a satisfactory therapeutic outcome. Additionally, the maintenance of lateral pharyngeal wall stability may be critical in sleep surgeries for subjects with OSA who show circumferential narrowing at the retropalatal level [17, 18].

Lateral pharyngoplasty, relocation pharyngoplasty, and ESP have been suggested to correct retropalatal circumferential narrowing in OSA. These surgical techniques are associated with greater AHI score improvement and more effectively widen the pharyngeal lumen by reducing collapse of the lateral pharyngeal wall during sleep [13–16]. Although relocation pharyngoplasty and ESP produced better therapeutic outcomes in OSA with lateral pharyngeal wall narrowing, most subjects experienced significant pain and dysphagia immediately following surgeries because they are invasive processes that undermine the palatopharyngeus muscle and can cause uncomfortable complications, such as oronasal reflux of liquid, or taste loss [13–18].

Soft-palate webbing flap pharyngoplasty appears to offer similar treatment benefits as relocation pharyngoplasty and ESP for OSA with lateral pharyngeal wall narrowing by maintaining posterior pillar tension. The original soft-palate webbing flap pharyngoplasty technique managed soft-palate narrowing and lateral pharyngeal wall collapse separately by creating two flaps from the redundant posterior pillar mucosal layers. This technique eliminated the postoperative possibility of a fibrotic soft palate, as reflected in the long-term maintenance of the widened oropharyngeal airway with sufficient lateral pharyngeal wall tension [19, 20]. Exposing the palatopharyngeus muscle at the upper part of the posterior pillar without requiring tonsillectomy has the advantage of manipulating the muscle away from other longitudinal pharyngeal muscles, thus preserving their function and avoiding the intra- and postoperative complications associated with tonsillectomy. We recommended soft-palate webbing flap pharyngoplasty to mild, moderate, and severe OSA with more than 50% narrowing in their lateral pharyngeal wall and tonsil enlargement, and we expected them to need greater tension to improve lateral pharyngeal wall collapse. We also expected soft-palate webbing flap pharyngoplasty to improve lateral pharyngeal wall collapse in OSA, and this procedure would be less invasive than relocation pharyngoplasty or ESP because tonsillectomy was not performed. Our data show that soft-palate webbing flap pharyngoplasty yielded a 42% success rate and a 71% response rate in OSA with lateral pharyngeal wall collapse. This technique provided a higher success rate in mild and moderate OSA than in severe OSA subjects, with 50% success in mild and 56% success in moderate OSA according to improved AHI score. Additionally, snoring intensity improved significantly. The success rate of soft-palate webbing flap pharyngoplasty was relatively lower than that of relocation pharyngoplasty and ESP in severe OSA with lateral pharyngeal wall narrowing.

Therefore, we suggest that the soft-palate webbing flap might be a favorable surgical technique to maintain pharyngeal tension in mild or moderate OSA with lateral pharyngeal wall narrowing and to provide good surgical outcomes to subjects with primary snoring with fewer complications. Our results are consistent with previous studies of surgical outcomes from soft-palate webbing flap pharyngoplasty [10, 20] that also concluded that it is an effective approach to improve sleep parameters, snoring intensity, and subjective symptoms such as breathing improvement, better sleep quality, and lower snoring volume in OSA. However, we provide a different surgical concept whether tonsillectomy need to be included for correction of lateral pharyngeal wall collapse. Bilateral tonsil hypertrophy might be one of the causes of lateral pharyngeal wall narrowing in subjects with OSA, and tonsillectomy may still be the most appropriate procedure among surgical options to improve lateral pharyngeal wall narrowing in a select group of subjects whose tonsil size is beyond grade 3 [17, 18, 30]. In these cases, lateral pharyngeal wall narrowing would not be effectively corrected if subjects with OSA did not undergo tonsillectomy. Therefore, the degree of tonsil hypertrophy and severity of OSA seem to be very critical in which surgical techniques to choose in OSA with lateral pharyngeal wall collapse. Subjects with OSA who underwent soft-palate webbing flap pharyngoplasty complained of minimal complications, and most were resolved within one month of surgery. However, we presume that the lateral pharyngeal wall of subjects after soft-palate webbing flap pharyngoplasty is not as strong as that after relocation pharyngoplasty or ESP, which both reposition the underlying muscular structures of the pharynx and palate to widen the lateral pharyngeal airway. We therefore suggest that soft-palate webbing flap pharyngoplasty may be particularly useful for primary snoring subjects and in mild or moderate OSA with greater than grade I circumferential narrowing at the retropalatal level and less than grade I tonsil enlargement. Subjects with OSA who have lateral pharyngeal wall narrowing and tonsil hypertrophy over grade III are not good candidates for soft-palate webbing flap pharyngoplasty. 

The recent trends in sleep surgery for OSA depend on accurate determination of upper airway collapse, and lateral pharyngeal wall collapse of recruited subjects was confirmed using sleep endoscopy in the present study. Recent study demonstrated a positional awake endoscopy can provide important surgical information with regard to level  and pattern of upper airway obstruction [31]. We did not perform a positional awake endoscopy to determine the site of occlusion in subjects included in this study and in fact, better research results may be obtained by measuring the degree of obstruction during sleep with DISE and comparing it with the results of awake endoscopy.

A major limitation of the present study was that the surgical outcome of soft-palate webbing flap pharyngoplasty was the result of combination with nasal surgeries, not single soft-palate webbing flap pharyngoplasty. However, it is difficult to recruit ubjects with only lateral pharyngeal wall collapse because the presence of multilevel upper airway collapse has been implicated in OSA pathophysiology. In addition, the study was retrospective and patients’ data did not include patient-reported outcome information such as behavioral, functional and quality of life. The clinical data of subjects were diverse and there was no control group in the present study. Therefore, the study was unable to estimate the therapeutic effect of soft-palate webbing flap pharyngoplasty precisely.

## Conclusion

The findings of this study suggest that soft-palate webbing flap pharyngoplasty may be a useful surgical option in subjects with OSA due to lateral pharyngeal wall collapse and provide improved therapeutic outcomes for patients with primary snoring, mild and moderate OSA with relatively low-grade tonsils.

## Data Availability

Data are available upon reasonable request.
